# Development and content validation of updated job competencies for Korean occupational therapists: a modified Delphi and focus group consensus study

**DOI:** 10.3352/jeehp.2026.23.16

**Published:** 2026-06-16

**Authors:** Byoung Gin Jeon, Seong-Youl Choi, Su-Yeon Park, Han-Som Kim, Jung-Jin Cha, Eun-Young Yoo, Hee-Jung Kim, Young-Geun Kim, Jeong-Sil Choi

**Affiliations:** 1 Department of Occupational Therapy, College of Health Science, Kangwon National University, Samcheok, Korea; 2 Department of Occupational Therapy, Faculty of Health and Medical Service, Daejeon Health University, Daejeon, Korea; 3 Department of Occupational Therapy, College of Health Sciences, Yonsei University, Wonju, Korea; 4 Department of Occupational Therapy, College of Medical and Science, Kosin Global University, Busan, Korea; 5 Department of Occupational Therapy, Daegu Health College, Daegu, Korea; 6 Department of Occupational Therapy, Field of Health and Medical, Chungbuk Health and Science University, Cheongju, Korea; Hallym University, Korea

**Keywords:** Certification, Focus groups, Group processes, Occupational therapists, Republic of Korea

## Abstract

**Purpose:**

This study aimed to analyze recent trends in occupational therapy, derive updated job competencies for Korean occupational therapists that reflect recent clinical changes, and confirm their validity.

**Methods:**

This 5-month expert consensus study, conducted from April to August 2024, used a 3-round online Delphi survey with 20 occupational therapy experts, followed by a focus group meeting with 10 experts, to refine and validate updated job competencies. Considering recent trends in occupational therapy, we adapted the U.S. National Board for Certification in Occupational Therapy practice analysis framework to the Korean context and collected expert feedback.

**Results:**

The Delphi panel identified several competency items that required contextualization within the Korean legal scope of practice, particularly items related to physical agent modalities. After the concluding focus group discussion, the superficial thermal agents item (D3-T1-K4) was excluded, and the electrotherapeutic modality item for swallowing disorders (D3-T1-K5) was revised; the remaining competencies were finalized.

**Conclusion:**

Updated job competencies for new occupational therapists were derived across 4 domains, 16 tasks, and 62 knowledge items. These competencies may support greater flexibility in international research and strengthen responses to the expansion of occupational therapists’ scope of practice in community settings. Therefore, the findings of this study are expected to be actively used in research, education, and practice.

## Graphical abstract

[Fig f2-jeehp-23-16]

## Introduction

### Background/rationale

As Korea’s population ages amid low birthrates, its jar-shaped demographic structure is gradually shifting toward an inverted pyramid. At the same time, the diversification of consumer needs for healthcare services requires occupational therapists to perform more specialized duties and assume more varied roles. The national examination system, which verifies the minimum standards, including knowledge and practical skills, required to work successfully as an occupational therapist, must be continuously reviewed to meet these social demands.

The duties and roles of occupational therapists are based on the Medical Service Technologists, etc. Act, and the scope of their duties has been further specified through the 2018 revision of the Enforcement Decree [1]. Accordingly, demand for occupational therapists in community settings is expanding beyond their traditional role in medical institutions. In addition to providing direct services to clients, occupational therapists are increasingly expected to work as community-based managers.

As a result of these changes, occupational therapists are expanding their employment settings from medical institutions, such as university hospitals, general hospitals, and rehabilitation hospitals, to non-medical institutions, such as schools, public health centers, dementia care centers, basic mental health welfare centers, welfare centers for persons with disabilities, community centers, and various rehabilitation and long-term care facilities [2]. In particular, the Act on Integrated Support for Community Care, Including Medical Care and Nursing Care, enacted in March 2024, is significant because it provides a future legal basis for visiting rehabilitation services. However, previous research on occupational therapists’ jobs has mainly focused on medical institutions, limiting its ability to reflect the recent diversification of occupational therapy jobs into non-medical institutions. Therefore, a new job analysis is needed to reflect the work of occupational therapists in both medical and non-medical institutions and to redefine job competencies for occupational therapists in Korea in line with these recent trends.

### Objectives

This study aimed to analyze recent trends in occupational therapy, derive updated job competencies for Korean occupational therapists that reflect recent clinical changes, and confirm their validity.

## Methods

### Ethics statement

The entire study process was approved by the Bioethics Committee of Kangwon National University under Institutional Review Board approval number KWNUIRB-2024-05-006-001. The study content was explained to participants in writing, and all participants provided written informed consent after confirming that they understood the study procedures.

### Study design

This mixed-methods expert consensus study combined a 3-round online Delphi survey with a concluding expert focus group meeting. The Delphi portion of the study was conducted and reported in accordance with the Guidance on Conducting and Reporting Delphi Studies (CREDES). To achieve expert consensus on the updated job competencies, 3 iterative Delphi rounds were conducted, followed by a final focus group meeting.

### Setting

**Fig. 1.**
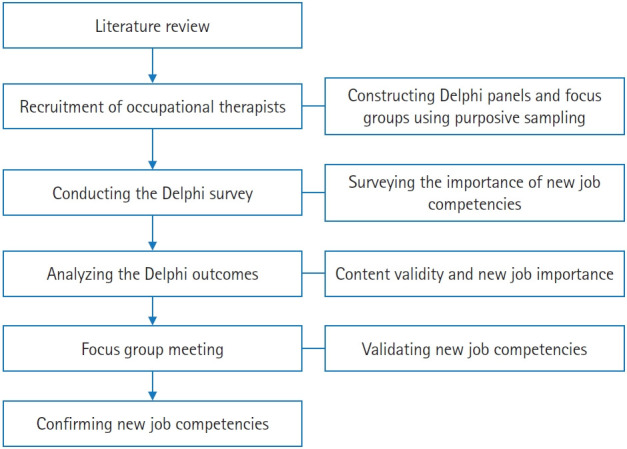
Flowchart of the study process.

This study involved interpreting the recently changed legal scope of practice, reviewing the literature, conducting Delphi surveys to confirm the validity of the updated job competencies among experts, and finalizing the competencies through an expert focus group meeting. The study lasted 5 months, from April to August 2024. The overall research process is illustrated in Fig. 1.

### Participants

#### Respondent group

A Delphi panel of 20 experts was selected to objectively evaluate the updated job competencies for occupational therapists. The inclusion criteria for panelists were as follows: (1) current managers overseeing occupational therapists in clinical settings and (2) professionals with at least 10 years of clinical experience who were capable of assessing the duties of new occupational therapists. Purposive sampling was used to ensure balanced representation across various institutions. These affiliations included tertiary hospitals, general hospitals, convalescent rehabilitation hospitals, long-term care hospitals, psychiatric hospitals, forensic hospitals, public health centers, dementia care centers, long-term care facilities, welfare facilities, pediatric therapy centers, quasi-governmental organizations, daycare centers, assistive technology centers, educational evaluation institutes, and the Road Traffic Authority.

#### Working group

The focus group was formed to ensure representative perspectives from both clinical practice and university education regarding the updated job competencies for occupational therapists. The group consisted of 7 members recommended by the Korean Association of Occupational Therapists and 3 members recommended by the Korean Association of Occupational Therapy Professors. All selected participants had at least 10 years of clinical or teaching experience. Purposive sampling was applied to ensure a balanced distribution of clinical fields, including hospitals, community settings, and pediatrics.

### Study size

For Delphi surveys, a panel of 20 to 30 participants is generally recommended when the target participants are patients or clinicians [3]. Therefore, in this study, a Delphi panel of 20 participants who met the minimum criteria was formed and surveyed. For focus group studies, theoretical agreement can generally be reached after collecting the opinions of 10 to 12 participants [4]. In this study, a focus group of 10 experts reached consensus on the clinical applicability of the updated job competencies for occupational therapists in Korea.

### Instrumentation

The initial item list for the Delphi survey was drafted based on a comprehensive literature review and the basic job analysis outcomes for occupational therapists provided by the National Board for Certification in Occupational Therapy (NBCOT). To systematically evaluate the competencies, each item was coded using a domain-task-knowledge framework (D#-T#-K#). The survey instrument used a 5-point Likert scale to quantitatively assess the clinical significance and validity of each job competency. The instrument also included open-ended sections that allowed the expert panel to provide qualitative professional opinions on each item.

### Protocol

The Delphi study was conducted iteratively over 3 planned rounds via email to systematically build expert consensus.

#### Round 1

Panelists rated the significance of the drafted job competencies using the 5-point Likert scale and provided open-ended qualitative feedback regarding the applicability of the items in the Korean clinical context.

#### Round 2

The questionnaire was revised based on the outcomes of the first round. Specific examples were added to the items to improve comprehension. To facilitate consensus, respondents were provided with the group median and interquartile range (IQR), excluding the bottom and top 25% of responses, from the first round, along with their own previous ratings. If a panelist’s second-round rating fell outside the group IQR, they were required to provide a specific rationale for their divergent response.

#### Round 3

A final re-rating phase was conducted to confirm the stability of the consensus. Following the 3 Delphi rounds, a concluding focus group meeting was held to comprehensively discuss the practical and legal implications of the agreed-upon items, such as resolving legal conflicts related to electrotherapy tasks. This process resulted in the final modification and confirmation of the updated job competencies.

### Data analysis/measurement

The significance of the updated job competencies among experts in occupational therapy was also assessed through the Delphi survey. These data were synthesized with the professional opinions of the focus group to determine the final job competencies.

### Bias

This study drew conclusions about updated job competencies by collecting the opinions of occupational therapy experts. Although the researchers attempted to obtain a balanced sample, they could not survey all occupational therapy experts. Therefore, the conclusions may be affected by the exclusion of potential participants who did not take part in the survey.

### Statistical methods

All study outcomes were analyzed using IBM SPSS ver. 26.0 (IBM Corp.), with the significance level set at α=0.05. The Delphi survey data were analyzed using the content validity ratio (CVR) and degree of consensus. According to Lawshe’s criterion, a CVR value of 0.42 or higher was considered acceptable for a panel of 20 experts; items with lower CVR values and/or low consensus were flagged for revision or discussion. The degree of consensus was calculated as 1−(Q3−Q1)/Mdn, where Q1 and Q3 represent the first and third quartiles, respectively, and Mdn represents the median. A larger value indicated greater consensus among the panelists [5].

## Results

### Participants

#### Delphi survey panel and response rate

The Delphi survey was conducted in 3 rounds, and the questionnaire was delivered to and retrieved from the experts via email. The response rate for all Delphi survey rounds was 100% (Table 1).

#### Characteristics of focus group participants

The expert group consisted of 10 participants: 7 clinicians in occupational therapy who were members of the Korean Association of Occupational Therapists and 3 professors from departments of occupational therapy who were members of the Korean Association of Occupational Therapy Professors (Table 2).

### Outcomes of analyzing the significance of updated job competencies among experts

Outcomes of the 1st and 2nd Delphi surveys: The first and second Delphi surveys used a 5-point Likert scale to assess the significance of each job competency, and additional professional opinions were provided for each item. The second Delphi questionnaire was developed based on the outcomes of the first round, and examples were added according to the expert panel’s opinions to improve comprehension of the questions. The median and interquartile range for each question, excluding the bottom and top 25% of all responses, were also presented with the first-round outcomes along with the respondents’ first-round ratings so that consensus among the expert group could be reviewed. If responses in the second survey were outside the interquartile range, respondents were asked to provide the reason.

As a result of the Delphi survey analysis, several items did not meet the CVR criterion in the first Delphi round. Of the 7 items with a first-round CVR of 0.40, 6 were revised based on expert feedback and increased to 0.50 in the second Delphi round, thereby meeting the criterion, whereas D4-T2-K2 remained at 0.40.

The round-to-round changes observed in some items were interpreted as reflecting revisions to item wording or the addition of examples based on participants’ qualitative feedback. This process was intended to improve item comprehension, and the expert opinions subsequently showed a gradual tendency toward convergence.

Therefore, these items were ultimately judged to be appropriate for inclusion in the occupational therapists’ job competencies (Table 3, Dataset 1).

### Confirmation of updated job competencies for occupational therapists

The researchers discussed the clinical significance and validity of the updated job competencies for new occupational therapists confirmed through the Delphi survey and focus group meeting. The Delphi panel and focus group consisted of occupational therapy education and clinical professionals. During this process, the researchers reviewed not only the job competencies but also the current criteria for the national examination for occupational therapists. Accordingly, occupational therapy clinical professionals emphasized the need for updated job competencies. Most job competencies were considered necessary for occupational therapists, but some items were identified as being less directly related to occupational therapy practice based on the professional opinions collected. Specifically, the experts discussed that (1) D3-T1-K4 was related to superficial thermal agents and D3-T1-K5 was related to electrotherapy for swallowing disorders, (2) these items could involve legal conflicts with other professions, and (3) occupational therapists’ work applies only to rehabilitation therapy for swallowing disorders. However, the experts agreed that occupational therapists currently use electrotherapy devices in dysphagia rehabilitation therapy in clinical practice and that knowledge of the indications, contraindications, precautions, and clinical applications of electrotherapy is necessary for occupational therapy practice. Therefore, D3-T1-K4, related to superficial thermal agents, was excluded, whereas D3-T1-K5, related to electrotherapy for swallowing disorders, was confirmed as a job competency after modification. The other items were finally agreed upon as updated job competencies after modifications and supplements (Table 4). The outcomes are provided as Supplements 1 and 2 because of the length of the study materials.

## Discussion

### Key results

Considering recent trends in occupational therapy, we reviewed the job applicability of the minimum job competencies of the NBCOT for new occupational therapists and confirmed their validity through Delphi surveys and focus group meetings with experts and related organizations. Through this process, Korea-specific job competencies were derived across 4 domains: “evaluation and assessment,” “analysis, interpretation, and planning,” “select and manage interventions,” and “competency and practice management.” These domains consisted of 3 tasks and 9 knowledge items in the “evaluation and assessment” domain; 3 tasks and 12 knowledge items in the “analysis, interpretation, and planning” domain; 6 tasks and 29 knowledge items in the “select and manage interventions” domain; and 4 tasks and 12 knowledge items in the “competency and practice management” domain.

### Interpretation

We examined recent international job trends in occupational therapy to derive updated job competencies for occupational therapists in Korea. The researchers selected NBCOT’s basic job analysis outcomes as a meaningful framework for job competencies. The basis for this selection was as follows: (1) The job competencies presented by NBCOT reflect all occupational therapy domains. (2) NBCOT’s job competency structure closely reflects work performance procedures according to the occupational therapy implementation framework.

Based on this evidence, NBCOT’s job competencies were analyzed and used to develop updated job competencies for new occupational therapists that reflect recent trends.

NBCOT completed a basic job analysis for Occupational Therapist Registered (OTR) professionals in 2022. The outcomes of this analysis were presented as domains, tasks, and knowledge items in the 2022 Comparative Analysis of Certification and Education Standards for the OTR, which identifies competencies essential for occupational therapists’ job performance [6]. To meet NBCOT certification requirements, candidates must complete and graduate from a training program approved by the Accreditation Council for Occupational Therapy Education or meet standard educational requirements [6]. Therefore, each knowledge item in NBCOT’s basic job analysis was compared with and analyzed against the educational standards of the Accreditation Council for Occupational Therapy Education (ACOTE). In this study, each job competency was coded using the D#-T#-K# format within the domain-task-knowledge framework, and updated job competencies for Korean occupational therapists were derived based on this framework.

### Comparison with previous studies

A review of 3 core studies on existing job changes and competency criteria for occupational therapists confirmed that the core jobs of occupational therapists consist of (1) assessment, (2) intervention planning and intervention, and (3) education and management. This finding is consistent with the job competency domains presented by NBCOT in 2022. In particular, the study by Jang et al. [7] has the advantage of presenting occupational therapists’ jobs in detail, making them easy to understand. Nevertheless, a limitation of that study was that it did not suggest a specific plan for educating or evaluating these job competencies. Therefore, in the present study, we built on the research of Jang et al. [7] and proposed updated job competencies as criteria for the national examination and for developing occupational therapist examination questions. Previous researchers have also methodologically revised and supplemented data from countries with advanced occupational therapy systems, and their findings do not appear to deviate substantially from NBCOT. Therefore, in this study, the latest version of the NBCOT job analysis (2022) was used to identify occupational therapy jobs. In particular, the ACOTE strategy, which links the curriculum to the knowledge units of job competencies, is expected to provide an advantage by facilitating future linkage with the Korea Accreditation Board for Occupational Therapy Education.

The job competencies reconstructed in this study based on the NBCOT framework and adapted to the Korean context represent a meaningful academic shift toward occupation-centered assessment, intervention, and management. However, this transition entails a fundamental shift away from the traditional skill-centered competencies currently used in clinical practice. Therefore, a transitional integration process will be necessary to successfully apply and adapt these updated competencies in both clinical and educational settings.

### Limitations

In this study, Korean job competencies were derived based on NBCOT. Although the clinical applicability of the updated job competencies was validated, further study is needed to examine their linkage with education. Finally, it will be necessary to investigate the use of the updated job competencies in actual clinical practice among occupational therapists nationwide.

### Generalizability

In this study, updated job competencies for Korea were derived based on NBCOT. These outcomes are significant because they strengthen the linkage with the global field of occupational therapy. Although NBCOT’s job competencies were developed for occupational therapists in the United States, they share structural similarities with competency frameworks adopted in other English-speaking countries [8]. Although not a direct comparison of job competencies, a study of student admission criteria in the United Kingdom has also been reported [9]. Considering these international trends, the outcomes of this study are expected to help increase the flexibility of international comparative analyses of job competencies for occupational therapists in Korea.

### Suggestions

Revised legislation since 2018 has significantly expanded opportunities for occupational therapists to enter non-medical institutions. However, although Korea has traditionally addressed occupational therapists’ jobs mainly in medical institutions, the United States defines occupational therapy jobs based on consensus across both medical and non-medical institutions. Therefore, NBCOT-defined jobs can provide a useful framework. The linkage between the ACOTE curriculum and NBCOT-defined jobs is considered a particular strength, and the NBCOT-based job competencies for Korean occupational therapists derived in this study can be integrated into university curricula to strengthen the connection among job competencies required for university education, national examinations, and clinical practice. Overall, future research should further strengthen the linkage between education and clinical practice.

### Conclusion

In this study, updated job competencies for occupational therapy were derived to reflect recent changes in the field of occupational therapy. These competencies consisted of 4 domains, 16 tasks, and 62 knowledge items. Because the competencies were derived based on job competencies for occupational therapists in the United States, they may support flexibility in international research. They are also expected to strengthen educational and practical responses to the expanding scope of occupational therapists’ work in community settings in light of legal changes in Korea.

## Figures and Tables

**Figure f2-jeehp-23-16:**
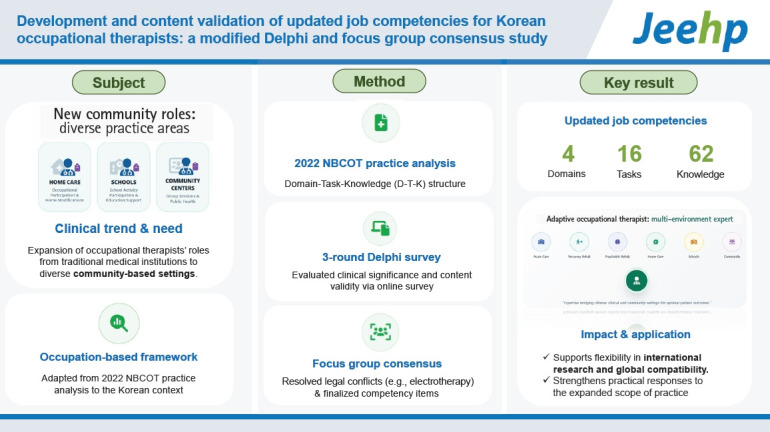


**Table 1.** Characteristics of the Delphi survey panel and response rate (n=20)

Characteristic	No. (%) or mean±SD	Delphi survey response rate (no., %)		
		1st	2nd	3rd
Gender				
Male	16 (80.0)	16 (100.0)	16 (100.0)	16 (100.0)
Female	4 (20.0)	4 (100.0)	4 (100.0)	4 (100.0)
Education level				
Associate’s degree	1 (5.0)	1 (100.0)	1 (100.0)	1 (100.0)
Bachelor’s degree	5 (25.0)	5 (100.0)	5 (100.0)	5 (100.0)
Master’s degree	12 (60.0)	12 (100.0)	12 (100.0)	12 (100.0)
Doctoral degree or higher	2 (10.0)	2 (100.0)	2 (100.0)	2 (100.0)
Age (yr)	41.70±6.94			
Clinical experience (yr)	14.32±7.52			

SD, standard deviation.

**Table 2.** General characteristics of the focus group (n=10)

Classification	Institution	Field
Representatives of institutions by field		
Clinician 1	ooo University Hospital	Tertiary general hospital
Clinician 2	ooo University Hospital	Tertiary general hospital
Clinician 3	ooo University Hospital	Tertiary general hospital
Clinician 4	ooo Hospital	Long-term care hospitals (adult)
Clinician 5	ooo Hospital	Long-term care hospitals (child)
Clinician 6	ooo Research Institute	Children’s centers
Clinician 7	ooo Dementia Care Center	Dementia care center
Member of the Korean Association of Occupational Therapy Professors		
Professor 1	oo University Department of Occupational Therapy	Korean Accreditation Board for Occupational Therapy Education
Professor 2	oo University Department of Occupational Therapy	Korean Association of Occupational Therapists
Professor 3	oo University Department of Occupational Therapy	Korean Association of Occupational Therapy Professors

**Table 3.** Outcomes of the Delphi survey on updated job competencies for occupational therapists

Domain	Task	Knowledge	1st CVR	2nd CVR	Outcomes of the 3rd Delphi survey		
					Mean±SD	CVR	Consensus
D1							
	T1						
		K1	0.80	1.00	4.70±0.57	0.90	0.95
		K2	0.90	1.00	4.90±0.31	1.00	1.00
		K3	0.80	1.00	4.85±0.37	1.00	1.00
		K4	0.60	0.70	4.50±0.51	1.00	0.78
	T2						
		K1	0.60	0.90	4.85±0.37	1.00	1.00
		K2	0.70	0.80	4.50±0.76	0.90	0.80
		K3	1.00	1.00	5.00±0.00	1.00	1.00
	T3						
		K1	0.50	0.60	4.20±0.52	0.90	0.94
		K2	0.90	1.00	4.85±0.37	1.00	1.00
D2							
	T1						
		K1	0.50	0.50	4.40±0.75	0.70	0.80
		K2	0.80	0.90	4.80±0.41	1.00	1.00
		K3	0.90	1.00	4.85±0.37	1.00	1.00
	T2						
		K1	0.60	0.80	4.35±0.49	1.00	0.75
		K2	**0.40**	0.50	3.80±0.70	0.50	0.94
		K3	0.80	0.90	4.75±0.44	1.00	0.95
		K4	0.70	0.80	4.30±0.57	0.90	0.75
		K5	0.70	0.70	4.30±0.57	0.90	0.75
	T3						
		K1	0.70	0.90	4.15±0.49	0.90	1.00
		K2	0.80	1.00	4.55±0.51	1.00	0.80
		K3	0.90	1.00	4.85±0.37	1.00	1.00
		K4	0.80	1.00	4.50±0.51	1.00	0.78
D3							
	T1						
		K1	0.60	0.70	4.45±0.51	1.00	0.75
		K2	0.60	0.90	4.20±0.52	0.90	0.94
		K3	0.70	0.80	4.05±0.69	0.60	0.94
		K4	1.00	1.00	3.45±0.89	**0.10**	0.75
		K5	0.50	0.60	3.35±0.93	**-0.20**	0.67
	T2						
		K1	0.90	1.00	4.35±0.49	1.00	0.75
		K2	0.50	0.50	4.70±0.47	1.00	0.80
		K3	0.80	0.90	4.10±0.64	0.70	0.94
		K4	0.90	1.00	4.30±0.73	0.70	0.75
		K5	0.60	0.80	4.50±0.51	1.00	0.78
		K6	**0.40**	0.50	4.55±0.51	1.00	0.80
		K7	0.80	0.90	4.65±0.59	0.90	0.80
		K8	0.70	0.80	4.10±0.79	0.50	0.69
		K9	0.70	0.70	4.15±0.59	0.80	0.94
		K10	0.70	0.90	4.10±0.79	0.50	0.69
	T3						
		K1	0.70	0.90	4.40±0.50	1.00	0.75
		K2	0.70	0.90	4.40±0.50	1.00	0.75
		K3	1.00	1.00	4.30±0.73	0.70	0.75
	T4						
		K1	0.60	0.90	4.25±0.44	1.00	0.94
		K2	0.70	1.00	3.95±0.69	0.50	0.94
		K3	0.70	1.00	4.45±0.51	1.00	0.75
		K4	**0.10**	**0.30**	4.65±0.49	1.00	0.80
	T5						
		K1	**0.10**	**0.20**	4.10±0.31	1.00	1.00
		K2	**0.00**	**0.10**	4.05±0.39	0.90	1.00
		K3	0.70	0.80	4.00±0.46	0.80	1.00
		K4	0.80	1.00	3.75±0.44	0.50	0.94
		K5	**0.40**	0.50	4.60±0.50	1.00	0.80
	T6						
		K1	**0.40**	0.50	3.75±0.44	0.50	0.94
		K2	0.60	0.70	3.60±0.50	**0.20**	0.75
D4							
	T1						
		K1	0.90	0.90	4.20±0.52	0.90	0.94
		K2	0.70	0.70	4.20±0.52	0.90	0.94
		K3	0.50	0.50	4.00±0.65	0.60	1.00
	T2						
		K1	**0.40**	0.50	3.70±0.47	**0.40**	0.75
		K2	**0.40**	**0.40**	4.30±0.47	1.00	0.75
		K3	0.50	0.60	4.65±0.49	1.00	0.80
		K4	0.50	0.60	4.25±0.55	0.90	0.75
	T3						
		K1	**0.40**	0.50	4.35±0.59	0.90	0.75
		K2	0.60	0.80	3.95±0.60	0.60	1.00
		K3	**0.30**	**0.40**	4.20±0.52	0.90	0.94
	T4						
		K1	0.60	0.90	4.35±0.59	0.90	0.75
		K2	0.80	0.90	4.25±0.72	0.70	0.75

Items below the CVR threshold are shown in bold. CVR, content validity ratio; SD, standard deviation.

**Table 4.** Summary of expert consensus: revised and excluded items

Job competencies	Domain-task-knowledge	Modification
Knowledge 4: Indications, contraindications, precautions and clinical application of superficial thermal agents (Example: dry whirlpool, hot packs, cryotherapy)	D3-T1-K4	Excluded
Knowledge 5: Indications, contraindications, precautions and clinical application of deep thermal, mechanical, and electrotherapeutic physical therapy for swallowing disorders (Example: transcutaneous and neuromuscular electrical stimulation, biofeedback)	D3-T1-K5	Revised

## Footnotes

**Data availability**
Data files are available from https://doi.org/10.7910/DVN/Y5YWTX Dataset 1. Raw data from the Delphi survey on updated job competencies for Korean occupational therapists.

## Supplementary materials

Supplement 1. New job competencies of Korean Occupational Therapists (English version).

Supplement 2. New job competencies of Korean Occupational Therapists (Korean version).

Supplement 3. Audio recording of the abstract.
