# Supplementary material for: Development and content validation of updated job competencies for Korean occupational therapists: a modified Delphi and focus group consensus study
Source: J Educ Eval Health Prof. 2026 Jun 16;23:16. doi: 10.3352/jeehp.2026.23.16 (PMC13472926; doi:10.3352/jeehp.2026.23.16)
Supplement: Supplementary file 1 [file jeehp-23-16-suppl1.docx]

| **Supplment 1. New Job Competencies of Korean Occupational Therapists** | | | **CODE** |
| --- | --- | --- | --- |
| **Domain 1: Evaluation and Assessment**  **Obtain information about factors that continuously affect occupational performance throughout the**  **occupational therapy process.** | | | **D1** |
|  | Task 1: Developmental and lifelong experiences; bodily functions and body structure; values, beliefs,  and spirituality; Understand the impact of identity on task performance | | D1-T1 |
|  |  | Knowledge 1: The impact of general development and aging on work performance, health, and  wellness throughout life | D1-T1-K1 |
|  |  | Knowledge 2: Expected patterns, progression, and prognosis associated with conditions  limiting task performance  (Example: signs and symptoms of disease, stages of disease, secondary complications) | D1-T1-K2 |
|  |  | Knowledge 3: The impact of body functions and structures on task performance  (Example: cognitive impairments, congenital anomalies) | D1-T1-K3 |
|  |  | Knowledge 4: The impact of life experiences and identity on work performance  (Example: history of trauma and adverse childhood events, socioeconomic status, gender, race,  culture, religion) | D1-T1-K4 |
|  | Task 2: Evaluate functional skills, roles, prioritized needs and desires, and performance environments to  evaluate client’s work performance | | D1-T2 |
|  |  | Knowledge 1: Resources and considerations to stay informed about client’s current status and  work performance  (Example: referrals, client records, developmental history, Individualized Education Program (IEP) plans, theoretical approach and frame of reference) | D1-T2-K1 |
|  |  | Knowledge 2: Administration, objectives, indications, advantages and limitations of standardized and non-standardized screening and assessment tools  (Example: criterion-referenced tests, norm-referenced tests, client and caregiver interviews, observation, occupational balance) | D1-T2-K2 |
|  |  | Knowledge 3: Internal and external factors that affect customer engagement  (Example: roles, habits, routines, and rituals, environmental context, family and social supports, medication side effects and interactions, mental health) | D1-T2-K3 |
|  | Task 3: Conduct activity analysis to determine the impact of task requirements, current conditions,  performance skills, and work environment | | D1-T3 |
|  |  | Knowledge 1: Task requirements based on environment or setting, task completion steps, and  task requirements | D1-T3-K1 |
|  |  | Knowledge 2: Personal and environmental factors that promote or inhibit task completion or  participation  (Example: compensation patterns, role of performance context, influence of current conditions) | D1-T3-K2 |
| **Domain 2: Analysis, Interpretation, and Planning**  **Establish client needs and priorities to develop and monitor intervention plans throughout the occupational**  **therapy process.** | | | **D2** |
|  | Task 1: Synthesis of evaluation & assessment results for service eligibility and customer-centered  intervention planning | | D2-T1 |
|  |  | Knowledge 1: Interpretation and analysis of quantitative assessment outcomes  (Example: statistical terminology, scoring, limitations of scores, client reactions to testing) | D2-T1-K1 |
|  |  | Knowledge 2: Interpretation of qualitative assessment outcomes  (Example: occupational profile, outcomes of needs assessment, results from interviews or observations) | D2-T1-K2 |
|  |  | Knowledge 3: Integrate and apply quantitative assessment outcomes and qualitative findings to guide  critical decisions and prioritize requirements | D2-T1-K3 |
|  | Task 2: Utilize a client-centered approach and therapeutic self-use to manage occupational therapy  services, working with clients, those around them, occupational therapy colleagues, and other professionals | | D2-T2 |
|  |  | Knowledge 1: Roles and responsibilities by interprofessional teams in customer care and service delivery (Example: referral to and consultation with other services, interprofessional communication methods, scope of practice) | D2-T2-K1 |
|  |  | Knowledge 2: Process for managing interprofessional intervention plans  (Example: Individualized Education Program (IEP) plans, discharge plans, transition plans, palliative and  hospice care plans) | D2-T2-K2 |
|  |  | Knowledge 3: Client-centric approach and considerations for coordinating occupational therapy services  (Example: therapeutic use of self, advocacy, frequency/duration of intervention, level of service delivery) | D2-T2-K3 |
|  |  | Knowledge 4: Factors used in collaboration with clients and families, teachers, caregivers, and other relevant bystanders to determine the prioritization of intervention objectives and activities  (Example: client-reported outcomes, level of support required, context of service delivery, progress  toward goals) | D2-T2-K4 |
|  |  | Knowledge 5: Strategies to enhance the ability of customers and those around them to navigate and interpret health-related information  (Example: caregiver training, education for client and caregiver, teaching-learning models, methods for  making health information accessible, informed decision-making, navigating challenges to professional expertise) | D2-T2-K5 |
|  | Task 3: Use clinical reasoning to monitor and modify ongoing intervention plans, approaches, context, and goals | | D2-T3 |
|  |  | Knowledge 1: Factors for determining and managing environments and activities to meet the goals  and objectives of interventions for individuals and groups | D2-T3-K1 |
|  |  | Knowledge 2: Strategies for monitoring effective interventions in individuals and groups  (Example: client-reported outcomes, reevaluation results, progress toward goals) | D2-T3-K2 |
|  |  | Knowledge 3: Clinical decision-making to evaluate, adapt, and prioritize objectives based on the client's response to the intervention  (Example: physiological changes, behavioral reactions, emotion regulation, developmental needs) | D2-T3-K3 |
|  |  | Knowledge 4: Precautions or contraindications related to the client's condition or recovery phase  (Example: postsurgical precautions, vital signs, laboratory values, suicidal ideation) | D2-T3-K4 |
| **Domain 3: Select and Manage Interventions**  **Select and implement interventions to promote healing and strengthen participation in work-based activities.** | | | **D3** |
|  | Task 1: Integration of preparatory techniques, activities and modalities as adjunct to interventions used to promote healing and enhance task-based activities | | D3-T1 |
|  |  | Knowledge 1: Considerations for selecting, preparing, and adapting intervention techniques to support optimal engagement and facilitate goal attainment  (Example: client condition, client safety) | D3-T1-K1 |
|  |  | Knowledge 2: Considerations for selecting, preparing, and adapting an intervention environment to support optimal engagement and facilitate goal attainment  (Example: ensuring privacy during telehealth sessions, equipment safety, line management) | D3-T1-K2 |
|  |  | Knowledge 3: Indications and precautions related to wound care and maintenance of skin integrity  (Example: stage of wound healing, prevention and management of pressure ulcers) | D3-T1-K3 |
|  |  | Knowledge 4: Indications, contraindications, precautions and clinical application of superficial thermal agents  (Example: dry whirlpool, hot packs, cryotherapy) | D3-T1-K4 |
|  |  | Knowledge 5: Indications, contraindications, precautions and clinical application of deep thermal, mechanical, and electrotherapeutic physical therapy for swallowing disorders  (Example: transcutaneous and neuromuscular electrical stimulation, biofeedback) | D3-T1-K5 |
|  | Task 2: Implement task-based strategies to support activities of daily living (ADLs), instrumental activities of daily living (IADLs), health care, rest and sleep, education, work, play, leisure and social participation across  the life span | | D3-T2 |
|  |  | Knowledge 1: Interventions to support leisure and play | D3-T2-K1 |
|  |  | Knowledge 2: Methods to grade activities, tasks, or techniques based on the level of development, the client's condition, the response to the intervention, and the client's needs | D3-T2-K2 |
|  |  | Knowledge 3: Considerations for facilitating individual and group participation in activities  (Example: group processes, group dynamics, group type and function) | D3-T2-K3 |
|  |  | Knowledge 4: Interventions to support cognitive, visual-motor, visual, and perceptual processing and sensory stimulation  (Example: sensory modulation, proprioceptive input, vestibular strategies, low vision strategies) | D3-T2-K4 |
|  |  | Knowledge 5: Compensatory and remedial strategies for managing cognitive and perceptual deficits or developmental disabilities  (Example: graded cueing, chaining, metacognition, memory aids) | D3-T2-K5 |
|  |  | Knowledge 6: Adaptive and preventive strategies to support optimal engagement in the work  (Example: use of technology/apps, joint protection, task simplification, energy conservation) | D3-T2-K6 |
|  |  | Knowledge 7: Intervention strategies and techniques used to promote oral motor skills for drinking, eating, and swallowing  (Example: hand-to-mouth patterns, mastication, adaptive utensils, positioning at mealtime) | D3-T2-K7 |
|  |  | Knowledge 8: Preoccupation, career, and transition services, options, and resources that support  strengths, interests, employment, and lifestyle goals throughout life | D3-T2-K8 |
|  |  | Knowledge 9: Strategies, techniques, and customer-centered training to facilitate functional transfers | D3-T2-K9 |
|  |  | Knowledge 10: Strategies to promote wellness and mental health  (Example: relaxation, sleep hygiene, addiction management) | D3-T2-K10 |
|  | Task 3: Select and implement interventions that improve sensory, motor, neurological and physiological status  to support task performance considering the client's condition and current stage of recovery | | D3-T3 |
|  |  | Knowledge 1: Methods to grade therapeutic exercise and conditioning programs, including indications  and precautions | D3-T3-K1 |
|  |  | Knowledge 2: Sensory and motor re-education, pain management, desensitization, edema reduction, and  scar management techniques | D3-T3-K2 |
|  |  | Knowledge 3: Techniques to promote improved postural stability, dynamic balance, body dynamics,  and breathing patterns through functional tasks | D3-T3-K3 |
|  | Task 4: Select, fabricate and modify orthotics and providing training in the use of orthotics & prosthetics to  help improve their functionality | | D3-T4 |
|  |  | Knowledge 1: Types and functions of immobilization, mobility, and restriction of orthotics for managing  specific conditions and congenital anomalies throughout life | D3-T4-K1 |
|  |  | Knowledge 2: Types and functions of prosthetics for upper limb and lower limb amputations | D3-T4-K2 |
|  |  | Knowledge 3: Anatomical, physiological, biomechanical, cognitive, and socioeconomic factors in the  selection, design, fabrication, and modification of orthotics, as well as the application of healing principles | D3-T4-K3 |
|  |  | Knowledge 4: Client-centered training methods for the safe and effective use of orthotic and prosthetic  devices | D3-T4-K4 |
|  | Task 5: Choose assistive technology options, adaptive devices, mobility aids, and other durable medical equipment to drive engagement | | D3-T5 |
|  |  | Knowledge 1: Factors related to the measurement, selection, appropriate monitoring and recommendation of seating systems, positioning devices, and mobility aids  (Example: equipment components, biomechanical considerations, usability and maintenance) | D3-T5-K1 |
|  |  | Knowledge 2: Types, traits, and characteristics of high-tech and low-tech assistive technologies and adaptive devices  (Example: interface options and processor parameters, equipment components) | D3-T5-K2 |
|  |  | Knowledge 3: Mobility options, vehicle adaptation and alternative devices to support community mobility participation  (Example: adapted driving controls, wheelchair tie downs, alternative transportation options, dynamic  mobile standers, navigation apps, dynamic mobile standers, navigation apps) | D3-T5-K3 |
|  |  | Knowledge 4: Customer factors influencing the successful use and maintenance of assistive technology options, adaptive devices, mobility aids, and other durable medical equipment (Example: developmental,  physical, functional, cognitive, and mental health status, prioritized needs) | D3-T5-K4 |
|  |  | Knowledge 5: Customer-centered education and training methods for the successful use and maintenance of assistive technology options, adaptive devices, mobility aids, and other durable medical equipment | D3-T5-K5 |
|  | Task 6: Recommend environmental modifications to support work engagement that includes client  needs, conditions, task demands, and context, while considering accessibility guidelines, standards, and laws | | D3-T6 |
|  |  | Knowledge 1: Ergonomics and universal design principles for the selection, recommendation, implementing features, and reasonable accommodations (RA) of workplace, home, virtual and public spaces | D3-T6-K1 |
|  |  | Knowledge 2: Processes and procedures for identifying, recommending, and applying modifications in  the workplace, home, virtual space, and public spaces | D3-T6-K2 |
| **Domain 4: Competency and Practice Management**  **Manage professional activities of oneself and related persons based on the basis of practice,**  **compliance regulations and standards to improve quality care** | | | **D4** |
|  | Task 1: Use evidence-based strategies and approaches to provide safe, effective, and efficient services  to individuals, groups, and populations | | D4-T1 |
|  |  | Knowledge 1: Methods to source, review, interpret, and critically appraise scholarly research to guide practice-relevant decision making  (Example: defining a clinical question, determining the clinical bottom line) | D4-T1-K1 |
|  |  | Knowledge 2: Methods for applying continuous quality improvement processes and procedures in the  delivery of occupational therapy services  (Example: program evaluation, outcome measures) | D4-T1-K2 |
|  |  | Knowledge 3: Evidence-based programming to improve health outcomes in populations  (Example: aging in place, fall prevention, health and wellness, community support groups) | D4-T1-K3 |
|  | Task 2: Integrate personal risk management techniques and practice environments to protect customers,  myself, employees, and others from injury or damage | | D4-T2 |
|  |  | Knowledge 1: Infection control procedures and prevention guidelines for reducing contaminant transmission  (Example: Personal Protective Equipment, isolation precautions, cleaning equipment) | D4-T2-K1 |
|  |  | Knowledge 2: Responses to adverse reactions, minor injuries, and emergencies  (Example: minor burns and cuts, seizures, diabetic reactions) | D4-T2-K2 |
|  |  | Knowledge 3: Preventive measures to minimize risk and promote safety  (Example: proper body mechanics, safety data sheet (SDS), standard operating procedures,  equipment maintenance, emergency preparedness, personal safety in the client’s environment) | D4-T2-K3 |
|  |  | Knowledge 4: Strategies and resources to prevent professional burnout  (Example: assessment of personal needs, self-advocacy regarding workload, stress management) | D4-T2-K4 |
|  | Task 3: Provide occupational therapy services in accordance with laws, regulations, and guidelines to protect consumers and meet applicable service environment requirements | | D4-T3 |
|  |  | Knowledge 1: Application of laws, regulations, and guidelines related to service delivery in the context of occupational therapy practice  (Example: health care legislation, accreditation organizations, licensing and credentialing) | D4-T3-K1 |
|  |  | Knowledge 2: Impact of occupational therapy service delivery guidelines and reimbursement policies | D4-T3-K2 |
|  |  | Knowledge 3: Accountability processes and procedures for justifying, tracking, and monitoring outcomes  (Example: relevant practice terminology, documentation guidelines) | D4-T3-K3 |
|  | Task 4: Participate in professional development and competency assessment activities related to job  roles, implementation setting, scope of work, and professional qualification criteria | | D4-T4 |
|  |  | Knowledge 1: Methods to check, document, and monitor service delivery competencies and professional development needs  (Example: competency self-assessment, mentoring, occupational based reports) | D4-T4-K1 |
|  |  | Knowledge 2: Application of ethical decision-making and professional behavior in accordance with the  code of ethics of the Korean Association of Occupational Therapists | D4-T4-K2 |
| : Job competencies redefined in conformity with the Korean situation | | | |
