# Supplementary material for: Development and content validation of updated job competencies for Korean occupational therapists: a modified Delphi and focus group consensus study
Source: J Educ Eval Health Prof. 2026 Jun 16;23:16. doi: 10.3352/jeehp.2026.23.16 (PMC13472926; doi:10.3352/jeehp.2026.23.16)
Supplement: Supplementary file 2 [file jeehp-23-16-suppl2.docx]

| **Supplment 2. 한국 작업치료사 신규 직무능력** | | | **CODE** |
| --- | --- | --- | --- |
| **도메인 1 : 평가 (Evaluation and Assessment)**  **작업치료 과정 전반에 걸쳐 지속적으로 작업수행에 영향을 미치는 요인에 대한 정보를 습득한다.** | | | **D1** |
|  | 과제 1 : 발달과 생애에 걸친 경험; 신체기능과 신체구조; 가치관, 신념, 영성; 정체성이 작업수행에 미치는 영향을 파악 | | D1-T1 |
|  |  | 지식 1 : 일반적인 발달과 노화가 전 생애에 걸쳐 작업수행, 건강 및 웰니스에 미치는 영향 | D1-T1-K1 |
|  |  | 지식 2 : 작업수행을 제한하는 조건과 관련된 예상되는 패턴, 진행 상황 및 예후(prognoses)  (예: signs and symptoms of disease, stages of disease, secondary complications ) | D1-T1-K2 |
|  |  | 지식 3 : 신체 기능과 구조가 작업수행에 미치는 영향  (예: cognitive impairments, congenital anomalies) | D1-T1-K3 |
|  |  | 지식 4 : 작업수행에서 삶의 경험과 정체성이 미치는 영향  (예: history of trauma and adverse childhood events, socioeconomic status, gender, race, culture, religion) | D1-T1-K4 |
|  | 과제 2 : 고객의 작업수행 평가를 위해 기능적 기술, 역할, 우선순위가 지정된 요구와 욕구, 수행 환경을 평가 | | D1-T2 |
|  |  | 지식 1: 고객의 현재 상태 및 작업수행에 대한 정보획득을 위한 자원 및 고려사항  (예: referrals, client records, developmental history, Individualized Education Program (IEP) plans, theoretical approach and frame of reference) | D1-T2-K1 |
|  |  | 지식 2: 표준화되고 비표준화된 선별 및 평가 도구의 관리(Administration), 목적, 적응증(indications), 장점 및 한계  (예: criterion-referenced tests, norm-referenced tests, client and caregiver interviews, observation, occupational balance) | D1-T2-K2 |
|  |  | 지식 3: 고객의 작업참여에 영향을 미치는 내부 및 외부 요인  (예: roles, habits, routines, and rituals, environmental context, family and social supports, medication side effects and interactions, mental health) | D1-T2-K3 |
|  | 과제 3 : 과제요구, 현재조건, 수행기술, 작업 환경의 영향을 결정하기 위해 활동분석을 수행 | | D1-T3 |
|  |  | 지식 1 : 환경 또는 셋팅에 따른 과제 요건, 과제 완료 단계 및 과제 요구 사항 | D1-T3-K1 |
|  |  | 지식 2 : 작업 완성 또는 참여를 촉진하거나 억제하는 개인적, 환경적 요인  (예: compensation patterns, role of performance context, influence of current conditions) | D1-T3-K2 |
| **도메인 2 : 분석, 해석 및 계획 (Analysis, Interpretation, and Planning)**  **작업치료 과정 전반에 걸쳐 중재 계획을 수립하고 모니터링하기 위해 고객의 요구와 우선순위를 확정한다.** | | | **D2** |
|  | 과제 1 : 서비스 적격성 결정과 고객 중심 중재 계획 수립을 위한 평가 결과 종합 | | D2-T1 |
|  |  | 지식 1 : 정량적(quantitative) 평가 결과의 해석과 분석  (예: statistical terminology, scoring, limitations of scores, client reactions to testing) | D2-T1-K1 |
|  |  | 지식 2 : 정성적(qualitative) 평가 결과의 해석  (예: occupational profile, outcomes of needs assessment, results from interviews or observations) | D2-T1-K2 |
|  |  | 지식 3 : 중요 의사결정을 가이드(guide)하고 요구사항의 우선순위 결정을 위한 정량적 평가 결과 및 정성적 발견을 통합 및 적용 | D2-T1-K3 |
|  | 과제 2 : 작업치료 서비스를 관리하기 위해 고객 중심 접근법과 치료적 자기 사용을 활용하여 고객, 고객의 주변인, 작업치료 동료 및 기타 전문가와 협력 | | D2-T2 |
|  |  | 지식 1 : 고객 관리 및 서비스 제공 시 전문가 팀(interprofessionalteams) 간의 역할과 책임  (예: referral to and consultation with other services, interprofessional communication methods, scope of practice) | D2-T2-K1 |
|  |  | 지식 2 : 전문가 간 중재 계획을 관리하기 위한 프로세스  (예: Individualized Education Program (IEP) plans, discharge plans, transition plans, palliative and hospice care plans) | D2-T2-K2 |
|  |  | 지식 3 : 작업치료 서비스 조정(coordinating)을 위한 고객 중심 접근 및 고려사항  (예: therapeutic use of self, advocacy, frequency/duration of intervention, level of service delivery) | D2-T2-K3 |
|  |  | 지식 4 : 고객 및 가족, 교사, 간병인 및 관련된 다른 주변인과 협력하여 중재 목표 및 활동의 우선순위 결정에 사용되는 요소  (예: client-reported outcomes, level of support required, context of service delivery, progress toward goals) | D2-T2-K4 |
|  |  | 지식 5 : 고객 및 관련 주변인의 건강 관련 정보 탐색 및 해석 능력을 강화하기 위한 전략  (예: caregiver training, education for client and caregiver, teaching-learning models, methods for making health information accessible, informed decision-making, navigating challenges to professional expertise) | D2-T2-K5 |
|  | 과제 3 : 임상적 추론을 사용하여 지속적 중재 계획, 접근법(approach), 환경(context), 목표를 모니터링하고 수정(modify) | | D2-T3 |
|  |  | 지식 1 : 개인 및 집단의 중재 목표와 목적을 충족하기 위한 환경 및 활동을 결정하고 관리하기 위한 요소 | D2-T3-K1 |
|  |  | 지식 2 : 개인 및 집단의 효과적인 중재를 모니터링하기 위한 전략  (예: client-reported outcomes, reevaluation results, progress toward goals) | D2-T3-K2 |
|  |  | 지식 3 : 중재에 대한 고객의 반응을 바탕으로 중재 계획을 평가하고 조정(adapting)하며 목표의 우선순위를 정하기 위한 임상적 의사결정  (예: physiological changes, behavioral reactions, emotion regulation, developmental needs) | D2-T3-K3 |
|  |  | 지식 4 : 고객의 상태 또는 회복 단계와 관련된 주의(Precautions) 또는 금기사항(contraindications)  (예: postsurgical precautions, vital signs, laboratory values, suicidal ideation) | D2-T3-K4 |
| **도메인 3 : 중재 선택과 관리 (Select and Manage Interventions)**  **치유를 촉진하고 작업기반 활동에 대한 참여를 강화하기 위한 중재를 선택하고 실행한다.** | | | **D3** |
|  | 과제 1 : 치유 촉진(promote healing) 및 작업기반 활동을 향상시키기 위해 사용하는 중재 보조수단(adjunct to interventions)인 준비 기술(preparatory techniques), 활동 및 수단(modalities)의 통합 | | D3-T1 |
|  |  | 지식 1 : 최적의 참여를 지원하고 목표 달성을 촉진하기 위해 중재 기법(technique)을 선택, 준비(preparing) 및 조정(adapting)하기 위한 고려사항  (예: client condition, client safety) | D3-T1-K1 |
|  |  | 지식 2 : 최적의 참여를 지원하고 목표 달성을 촉진하기 위해 중재 환경(environment)을 선택, 준비(preparing) 및 조정(adapting)하기 위한 고려사항  (예: ensuring privacy during telehealth sessions, equipment safety, line management ) | D3-T1-K2 |
|  |  | 지식 3 : 상처 관리 및 피부 무결성(skin integrity) 유지와 관련된 적응증, 주의사항  (예: stage of wound healing, prevention and management of pressure ulcers) | D3-T1-K3 |
|  |  | 지식 4 : 표재온열요법(superficial thermal agents)의 적응증, 금기사항, 주의사항 및 임상적용  (예: dry whirlpool, hot packs, cryotherapy ) | D3-T1-K4 |
|  |  | 지식 5 : 삼킴장애를 위한 심부열, 기계, 전기치료 물리 요법의 적응증, 금기사항, 주의사항 및 임상적용  (예: transcutaneous and neuromuscular electrical stimulation, biofeedback) | D3-T1-K5 |
|  | 과제 2 : 전 생애에 걸쳐 일상생활활동(ADL), 수단적일상생활활동(IADL), 건강관리, 휴식 및 수면, 교육, 일, 놀이, 여가 및 사회참여를 지원하기 위한 작업기반 전략의 실행 | | D3-T2 |
|  |  | 지식 1 : 여가 및 놀이 지원을 위한 중재 | D3-T2-K1 |
|  |  | 지식 2 : 발달 수준, 고객의 상태, 중재의 반응, 고객의 요구를 기준으로 활동, 과제 또는 기법을 등급화하는 방법 | D3-T2-K2 |
|  |  | 지식 3 : 활동에 대한 개인 및 그룹의 참여 촉진을 위한 고려 사항  (예: group processes, group dynamics, group type and function) | D3-T2-K3 |
|  |  | 지식 4 : 인지적, 시각-운동적, 시각적, 지각적 처리와 감각적 각성을 지원하기 위한 중재  (예: sensory modulation, proprioceptive input, vestibular strategies, low vision strategies) | D3-T2-K4 |
|  |  | 지식 5 : 인지 및 지각 결손 또는 발달 장애 관리를 위한 보상(Compensatory) 및 회복(remedial) 전략  (예: graded cueing, chaining, metacognition, memory aids) | D3-T2-K5 |
|  |  | 지식 6 : 작업에 대한 최적의 참여를 지원하기 위한 적응(Adaptive) 및 예방(preventive) 전략  (예: use of technology/apps, joint protection, task simplification, energy conservation) | D3-T2-K6 |
|  |  | 지식 7 : 마시기, 먹기, 삼킴을 위한 구강 운동 기술을 촉진하기 위해 사용되는 중재 전략 및 기법  (예: hand-to-mouth patterns, mastication, adaptive utensils, positioning at mealtime) | D3-T2-K7 |
|  |  | 지식 8 : 전 생애에 걸쳐 강점, 흥미, 고용 및 라이프스타일의 목표를 지지(supporting)하는 직업 전, 직업, 전환 서비스, 옵션 및 자원 | D3-T2-K8 |
|  |  | 지식 9 : 기능 전이(functional transfers)를 촉진하기 위한 전략, 기법 및 고객 중심 교육 | D3-T2-K9 |
|  |  | 지식 10 : 웰니스와 정신 건강을 증진하기 위한 전략  (예: relaxation, sleep hygiene, addiction management) | D3-T2-K10 |
|  | 과제 3 : 고객의 상태와 현재 회복단계를 고려하여 작업수행을 지원하기 위한 감각, 운동, 신경학 및 생리학적 상태를 개선하는 중재를 선택하고 실행 | | D3-T3 |
|  |  | 지식 1 : 적응증 및 주의사항을 포함한 치료적 운동 및 컨디셔닝(conditioning) 프로그램의 등급을 매기는 방법 | D3-T3-K1 |
|  |  | 지식 2 : 감각 및 운동 재교육, 통증 관리, 탈감각(desensitization), 부종 감소 및 흉터 관리(scar management) 기술 | D3-T3-K2 |
|  |  | 지식 3 : 기능적 과제를 통해 향상된 자세 안정성, 동적 균형, 신체 역학 및 호흡 패턴을 촉진하기 위한 기술 | D3-T3-K3 |
|  | 과제 4 : 보조기를 선택, 제작 및 수정하고 보조기 및 의지 (orthotic & prosthetic) 사용에 대한 교육을 제공하여 기능 향상 지원 | | D3-T4 |
|  |  | 지식 1 : 생애 전반에 걸친 특정 상태 및 선천적 이상(congenital anomalies)을 관리하기 위한 보조기의 고정성(immobilization), 가동성, 제한성(restriction)의 유형 및 기능 | D3-T4-K1 |
|  |  | 지식 2 : 상지 절단 및 하지 절단용 의지의 종류 및 기능 | D3-T4-K2 |
|  |  | 지식 3 : 보조기 선택, 설계, 제작 및 수정의 해부학적, 생리학적, 생체역학적, 인지적, 사회경제적 요소와 치유 원리(healing principles)의 적용 | D3-T4-K3 |
|  |  | 지식 4 : 보조기 및 의지 장치의 안전하고 효과적인 사용을 위한 고객 중심의 교육훈련 방법 | D3-T4-K4 |
|  | 과제 5 : 보조공학 기술 옵션, 적응형 장치, 이동 보조장치 및 기타 내구성이 뛰어난 의료장비를 선택하여 작업 참여를 촉진 | | D3-T5 |
|  |  | 지식 1 : 착석 시스템, 포지셔닝 장치 및 이동 보조 장치의 측정, 선택, 적합성 모니터링 및 수정 권장과 관련된 요소  (예: equipment components, biomechanical considerations, usability and maintenance) | D3-T5-K1 |
|  |  | 지식 2 : 하이테크 및 로우테크 보조공학 기술 및 적응형(adaptive) 디바이스의 종류, 특성 및 특징  (예: interface options and processor parameters, equipment components) | D3-T5-K2 |
|  |  | 지식 3 : 지역사회 이동 참여지원을 위한 이동 옵션, 차량 적응 및 보조(alternative) 장치  (예: adapted driving controls, wheelchair tie downs, alternative transportation options, dynamic mobile standers, navigation apps, dynamic mobile standers, navigation apps) | D3-T5-K3 |
|  |  | 지식 4 : 보조공학 기술 옵션, 적응형 장치(adaptive devices), 이동 보조 장치 및 기타 내구성이 뛰어난 의료 장비의 성공적인 사용 및 유지 관리에 영향을 미치는 고객 요인  (예: developmental, physical, functional, cognitive, and mental health status, prioritized needs) | D3-T5-K4 |
|  |  | 지식 5 : 보조공학 기술 옵션, 적응형 장치(adaptive devices), 이동 보조 장치 및 기타 내구성이 뛰어난 의료 장비의 성공적인 사용 및 유지 관리를 위한 고객 중심 교육 및 훈련 방법 | D3-T5-K5 |
|  | 과제 6 : 접근성 지침(accessibility guidelines), 표준 및 법률을 고려하면서 고객의 요구와 상태, 과제요구 및 맥락(context)을 포함하는 작업 참여를 지원하기 위해 환경수정을 추천 | | D3-T6 |
|  |  | 지식 1 : 작업공간(workplace), 가정, 가상(virtual)공간 및 공공(public)공간의 선별, 추천, 기능 구현(implementing features), 합리적 조정/변경(RA, reasonable accommodations)을 위한 인체공학 및 유니버셜 디자인의 원리 | D3-T6-K1 |
|  |  | 지식 2 : 작업공간(workplace), 가정, 가상공간 및 공공공간의 수정사항 확인, 권고 및 적용을 위한 프로세스(Processes) 및 절차(procedures) | D3-T6-K2 |
| **도메인 4 : 역량 및 실행 관리 (Competency and Practice Management)**  **양질의 관리(care) 향상을 위해 실행의 근거, 준수 규정 및 표준을 기반으로 자신과 관련인의 전문가적 활동 관리(manage)** | | | **D4** |
|  | 과제 1 : 개인, 그룹 그리고 모집단과 관련된 안전하고 효과적이며 효율적인 서비스를 제공하기 위해 근거기반 전략과 접근방식을 사용 | | D4-T1 |
|  |  | 지식 1 : 실행 관련(practice-relevant) 의사결정을 안내하기 위한 학술 연구의 출처, 검토, 해석 및 비판적 평가 방법  (예: defining a clinical question, determining the clinical bottom line) | D4-T1-K1 |
|  |  | 지식 2 : 작업치료 서비스 제공의 지속적인 품질향상 프로세스 및 절차를 적용하기 위한 방법  (예: program evaluation, outcome measures) | D4-T1-K2 |
|  |  | 지식 3 : 모집단(population)의 건강 결과를 개선하기 위한 근거 기반 프로그래밍  (예: aging in place, fall prevention, health and wellness, community support groups) | D4-T1-K3 |
|  | 과제 2 : 고객, 자신, 직원 및 기타 사람들을 부상 또는 손상으로부터 보호하기 위한 개인의 위험 관리 기술과 실천 환경의 통합 | | D4-T2 |
|  |  | 지식 1 : 감염관리 절차 및 오염물질(contaminants) 전파 감소를 위한 예방지침  (예: Personal Protective Equipment, isolation precautions, cleaning equipment) | D4-T2-K1 |
|  |  | 지식 2 : 부작용(Responses to adverse reactions), 경미한 부상 및 응급 상황에 대한 대응  (예: minor burns and cuts, seizures, diabetic reactions) | D4-T2-K2 |
|  |  | 지식 3 : 위험을 최소화하고 안전을 증진하기 위한 예방 조치  (예: proper body mechanics, safety data sheet (SDS), standard operating procedures, equipment maintenance, emergency preparedness, personal safety in the client’s environment) | D4-T2-K3 |
|  |  | 지식 4 : 전문가의 소진(professional burnout)을 방지하기 위한 전략 및 리소스  (예: assessment of personal needs, self-advocacy regarding workload, stress management) | D4-T2-K4 |
|  | 과제 3 : 소비자를 보호하고 적용 가능한 서비스 환경 관련 요구 사항을 충족하기 위해 법, 규정, 지침에 따라 작업치료 서비스를 제공 | | D4-T3 |
|  |  | 지식 1 : 작업치료 실행 환경에 따른 서비스 전달 관련 법, 규정, 지침의 적용  (예: health care legislation, accreditation organizations, licensing and credentialing) | D4-T3-K1 |
|  |  | 지식 2 : 작업치료 서비스 제공 지침과 배상 정책(reimbursement policies)의 영향 | D4-T3-K2 |
|  |  | 지식 3 : 결과를 정당화(justifying), 추적 및 모니터링하기 위한 책임(Accountability) 프로세스 및 절차  (예: relevant practice terminology, documentation guidelines) | D4-T3-K3 |
|  | 과제 4 : 직무 역할, 실행 환경, 업무 범위 및 전문 자격 기준과 관련된 전문성 개발 및 역량 평가 활동에 참여 | | D4-T4 |
|  |  | 지식 1 : 서비스 제공 역량과 전문성 개발 필요성을 점검, 문서화, 모니터링하는 방법  (예: competency self-assessment, mentoring, occupational based reports) | D4-T4-K1 |
|  |  | 지식 2 : (사)대한작업치료사협회 윤리강령에 따른 윤리적 의사결정 및 전문가적 행동의 적용 | D4-T4-K2 |
| : 한국 실정에 맞게 재정의한 직무능력 | | | |
